# Exploring Bacterial Diversity in Hospital Environments by GS-FLX Titanium Pyrosequencing

**DOI:** 10.1371/journal.pone.0044105

**Published:** 2012-08-29

**Authors:** Margarita Poza, Carmen Gayoso, Manuel J. Gómez, Soraya Rumbo-Feal, María Tomás, Jesús Aranda, Ana Fernández, Germán Bou

**Affiliations:** 1 Microbioloy Department, Biomedical Research Institute-University Hospital, A Coruña, Spain; 2 Sequencing and Bioinformatics Department, Astrobiology Center INTA-CSIC, Madrid, Spain; University of Vienna, Austria

## Abstract

Understanding microbial populations in hospital environments is crucial for improving human health. Hospital-acquired infections are an increasing problem in intensive care units (ICU). In this work we present an exploration of bacterial diversity at inanimate surfaces of the ICU wards of the University Hospital A Coruña (Spain), as an example of confined hospital environment subjected to selective pressure, taking the entrance hall of the hospital, an open and crowded environment, as reference. Surface swab samples were collected from both locations and recovered DNA used as template to amplify a hypervariable region of the bacterial 16S rRNA gene. Sequencing of the amplicons was performed at the Roche 454 Sequencing Center using GS-FLX Titanium procedures. Reads were pre-processed and clustered into OTUs (operational taxonomic units), which were further classified. A total of 16 canonical bacterial phyla were detected in both locations. Members of the phyla Firmicutes (mainly *Staphylococcus* and *Streptococcus*) and Actinobacteria (mainly Micrococcaceae, Corynebacteriaceae and Brevibacteriaceae) were over-represented in the ICU with respect to the Hall. The phyllum Proteobacteria was also well represented in the ICU, mainly by members of the families Enterobacteriaceae, Methylobacteriaceae and Sphingomonadaceae. In the Hall sample, the phyla Proteobacteria, Bacteroidetes, Deinococcus-Thermus and Cyanobacteria were over-represented with respect to the ICU. Over-representation of Proteobacteria was mainly due to the high abundance of Enterobacteriaceae members. The presented results demonstrate that bacterial diversity differs at the ICU and entrance hall locations. Reduced diversity detected at ICU, relative to the entrance hall, can be explained by its confined character and by the existence of antimicrobial selective pressure. This is the first study using deep sequencing techniques made in hospital wards showing substantial hospital microbial diversity.

## Introduction

Metagenomics advances due to the increased availability of high throughput platforms for DNA sequencing and associated bioinformatics software are revolutionizing our knowledge about microbial communities. Direct studies of microorganisms that are difficult to culture (as much as 99% of the microorganisms living in a natural environment) may now be performed by these modern techniques. Prokaryotic 16S ribosomal RNA genes (18S rRNA genes in the case of eukarya) have been commonly used for studying biodiversity since they are universally distributed in all cellular organisms and allow the identification and comparison of microorganisms present in a wide variety of environmental samples [1]–[3]. Many authors have used this approach to explore 16S rRNA diversity in natural samples [4]–[6]. Pyrosequencing techniques have the great advantage of allowing direct sequencing of 16S rRNA collections without the need of the cloning step. The obtained collection of 16S rRNA sequences can then be compared against reference databases such as GenBank and RDP (Ribosomal Database Project, http://rdp.cme.msu.edu/), which include more than 800,000 16S rRNA sequences, with the aim of having them classified.

In the last decade, many studies have been performed to describe microbial populations of samples ranging from the abyssal sea floor [7] or the polar desert [8] to the water and air of a hospital therapy pool [9]. In 2004, whole genome shotgun sequencing techniques were applied to microbial populations collected from sea water samples of the Sargasso Sea [10]. Since then, many studies describing microbial diversity have been done applying deep sequencing technologies from different samples as marine environments [11], [12] or forest and grassland soils [6]. In only one decade it became clear that most of the bacteria isolated from natural environments were not represented in culture collections. Moreover, recent studies directed to describe microbial populations living in the human body, as the human skin microbiome characterized by Grice *et al*., [4] or the oral or gastrointestinal microbiome, explored by several groups using deep sequencing technologies [13]–[19], have revolutionized our understanding about the substantial diversity of microorganisms co-habiting with us. The application of new technologies has promoted the discovery that biodiversity is much more complex than traditional culture methods indicated in the past.

Hospital-acquired infections are the sixth leading cause of death in the United States and similar data have been reported from Europe [20], being an increasing problem in intensive care units (ICU), where the patients are more susceptible to colonization and the organisms are often more resistant to antimicrobial agents than in other environments. Since the 80's, infectious disease specialists have recognized that ICU patients acquire nosocomial infections at a much higher rate than patients elsewhere in the hospital [21]. In these confined areas, lower respiratory tract and bloodstream infections happen to be the most lethal; however, urinary tract infections are the most common. Infections caused by gram-negative bacteria have features that are of particular concern since these organisms are highly efficient at up-regulating or acquiring genes that code for mechanisms of antibiotic drug resistance, especially in the presence of antibiotic selection pressure. Most common nosocomial pathogens may well survive or persist on surfaces for a long time, what could explain their survival in ICU wards [22]. Gram-positive bacteria, such as *Enterococcus* spp. (including VRE), *Staphylococcus aureus* (including MRSA), *Clostridium difficile* or *Streptococcus pyogenes*, and many gram-negative bacteria, such as *Acinetobacter* spp., *Klebsiella* spp., *Pseudomonas aeruginosa*, *Serratia marcescens*, or *Shigella* spp., may indeed survive for months on dry surfaces. A few others, such as *Bordetella pertussis*, *Haemophilus influenzae*, *Proteus vulgaris*, or *Vibrio cholerae*, however, persist only for days. In the present work we describe for the first time bacterial diversity in hospital environments using modern deep sequencing technologies. We present an exploration of the bacterial diversity at the ICU wards of the University Hospital A Coruña (Spain), as an example of confined hospital environment subjected to selective pressure, using 16S rRNA gene hypervariable region pyrosequencing. As a contrasting reference, we have also studied bacterial diversity at an open and crowded environment, the entrance hall of the hospital.

## Results

### Sequence Data

A fragment of the V7–V9 hypervariable region of the bacterial 16S rRNA gene, around 450–480 base pairs long, was amplified by PCR with primers *mp16S2f* and *mp16S2r* from genomic DNA obtained from surface swab samples collected at ICU ward and at the entrance hall of the University Hospital A Coruña (Spain). The two collections of amplicons were subjected to 454 pyrosequencing at the Roche Sequencing Center. The number of reads obtained was 501707 and 346427 for ICU and Hall samples, respectively (Table 1).

**Table 1:** Total number of reads accumulated for each sample in the original data sets and after filtering and classification.

Sample	Original numberof reads	Number of reads afterpre-processing	Number of reads aftersub-sampling	Number of reads classifiedby Mothur
ICU	501707	95104 (18%)	95104 (18%)	95103 (18%)
Hall	346427	99952 (28%)	95104 (27%)	95071 (27%)

### Sequence Pre-processing

The analysis of the read length distributions indicated that the modal length was 458 nt for the ICU sample and 457 for the Hall one. However, a significant number of reads had shorter lengths. Reads were filtered and trimmed as described in Materials and Methods, resulting in datasets containing around 42% and 45% of the original reads for the ICU and Hall samples, respectively. Their alignment, against the Core Set reference alignment of the Greengenes database, and subsequent screening, to optimize the number of them covering the same sequence space, resulted in datasets containing around 19% and 30% of the original reads. Reads were then grouped using a pseudo-single linkage algorithm, to reduce the impact of pyrosequencing noise, and screened with UCHIME to discover and eliminate chimeric sequences. This last step eliminated 7635 chimeric sequences from both samples, resulting in datasets containing 18% and 28% of the original reads for the ICU and Hall samples, respectively (Table 1). Finally, datasets were sub-sampled to normalize the number of reads in each sample (Table 1).

### OTU Detection

**Figure 1:**
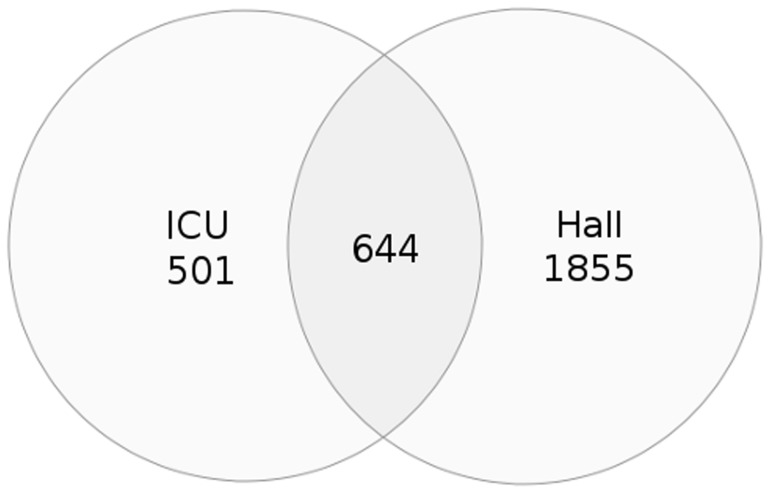
Venn diagram representation of shared richness at a distance of 0.03.

**Figure 2:**
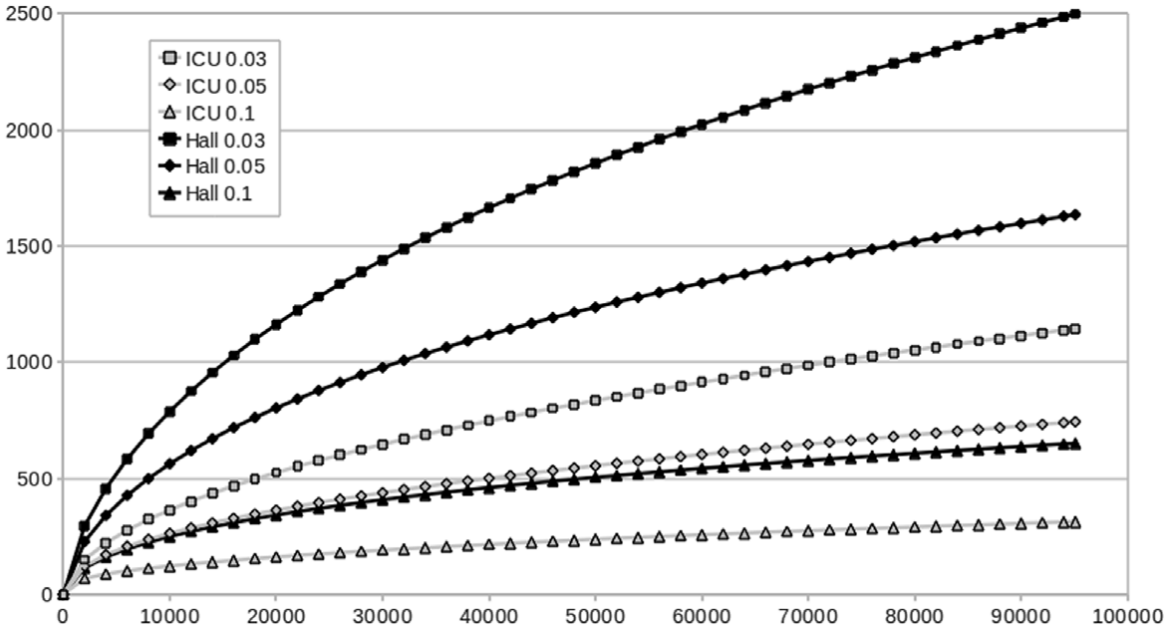
Rarefaction curves for the ICU and Hall samples at distances of 0.03, 0.05 and 0.1.

Operational taxonomic units (OTUs) were defined by clustering pre-processed, grouped and normalized reads as described in Materials and Methods. The number of OTUs detected at a distance of 0.03, which is assumed to correspond to the species level, was 1145 for the ICU sample and 2499 for Hall sample (Table 2). The total number of OTUs observed in the two samples was 3000. The number of OTUs that were observed as shared between the ICU and Hall samples was 644 (Fig.1). The Ace and Chao1 estimates of community richness at a distance of 0.03 had values of 2798 and 2117, respectively, for the ICU sample, and 5043 and 4279 for the Hall sample (Table 2), suggesting that diversity at species level could still be up to two times higher than observed, approximately, and that the Hall sample contained a higher diversity than the ICU sample, in terms of species richness. In agreement with this observation, the shape of rarefaction curves calculated at 0.03, 0.05 and 0.1 distances indicate a stronger trend to saturation for the ICU sample than for the Hall sample (Fig. 2). It should be noted that, to prevent the obliviation of rare phylotypes, singleton OTU removal was not included in the data processing pipeline. Therefore, observed and estimated richness values could be overestimated. However, this possibility would not be an explanation for the difference in richness observed for the Hall and ICU samples, since a test in which singleton OTUs were discarded yielded the same result: Hall was twice as diverse as ICU, in terms of species richness (data not shown).

**Table 2:** Observed and predicted richness.

Sample	Reads	Clustering distance								
		0.03			0.05			0.1		
		OTU	ACE	Chao1	OTU	ACE	Chao1	OTU	ACE	Chao1
ICU	95104	1145	2798	2117	744	1729	1343	314	750	516
Hall	95104	2499	5043	4279	1636	3206	2696	651	1203	1017

Observed richness is presented, for each location, as the number of OTUs defined at clustering distances of 0.03, 0.05 and 0.1. Predicted richness is presented as the values of the Ace and Chao1 diversity estimators, at the same clustering distances.

### OTU Classification

**Figure 3:**
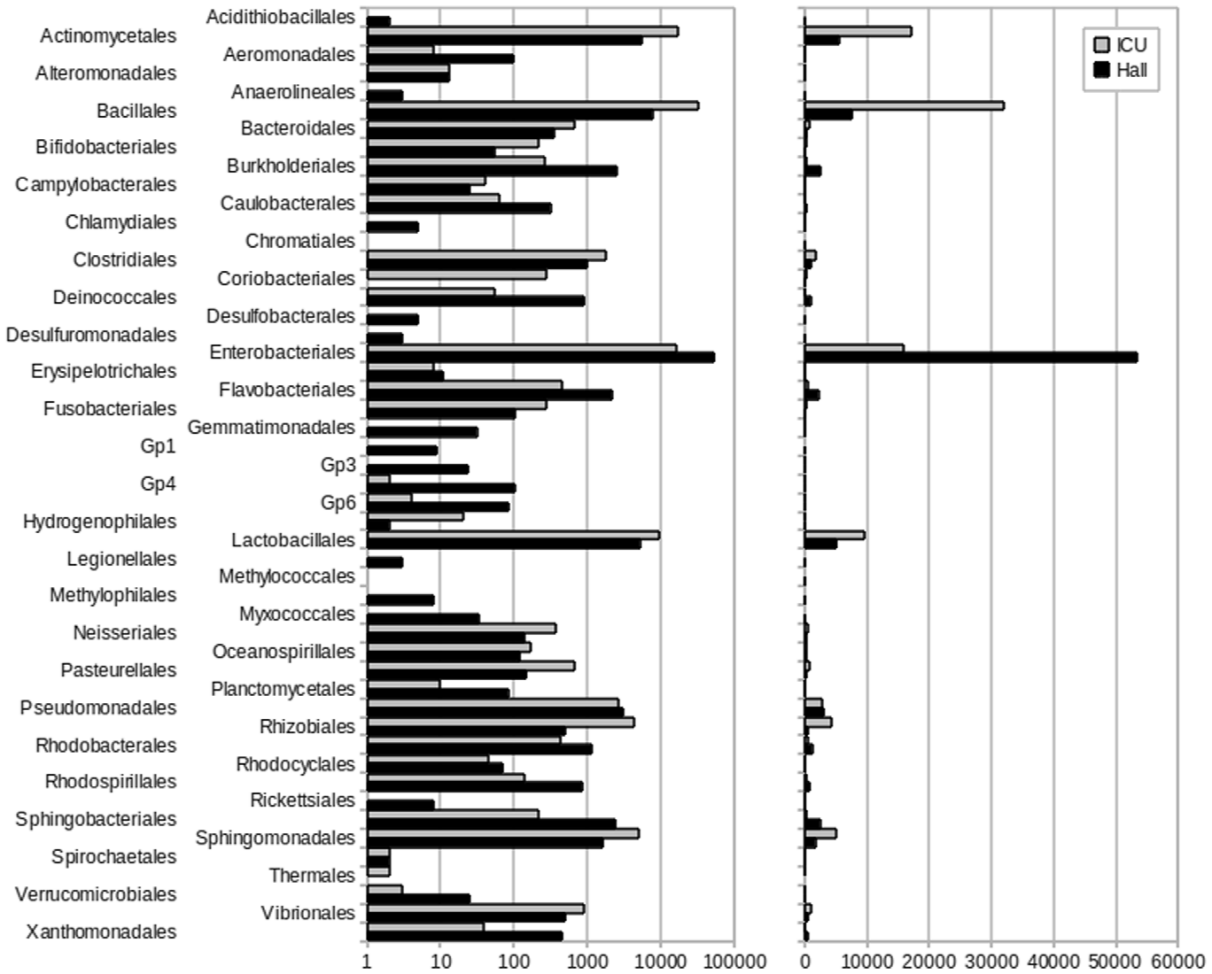
Normalized abundance of reads associated with taxonomic nodes at the order level, expressed as the number of occurrences in 100000 reads. Abundances are shown in logarithmic (left panel) and decimal (right panel) scales.

**Figure 4:**
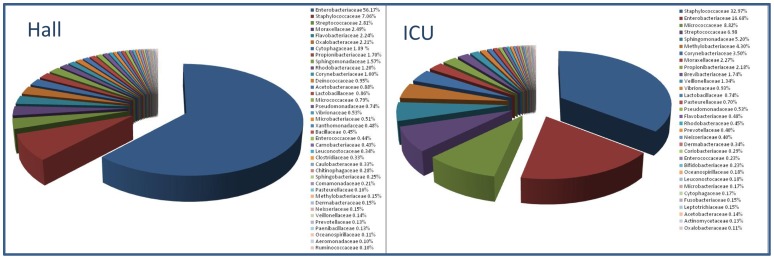
Schematic representation of the relative abundance of families described in Hall and ICU samples.

**Figure 5:**
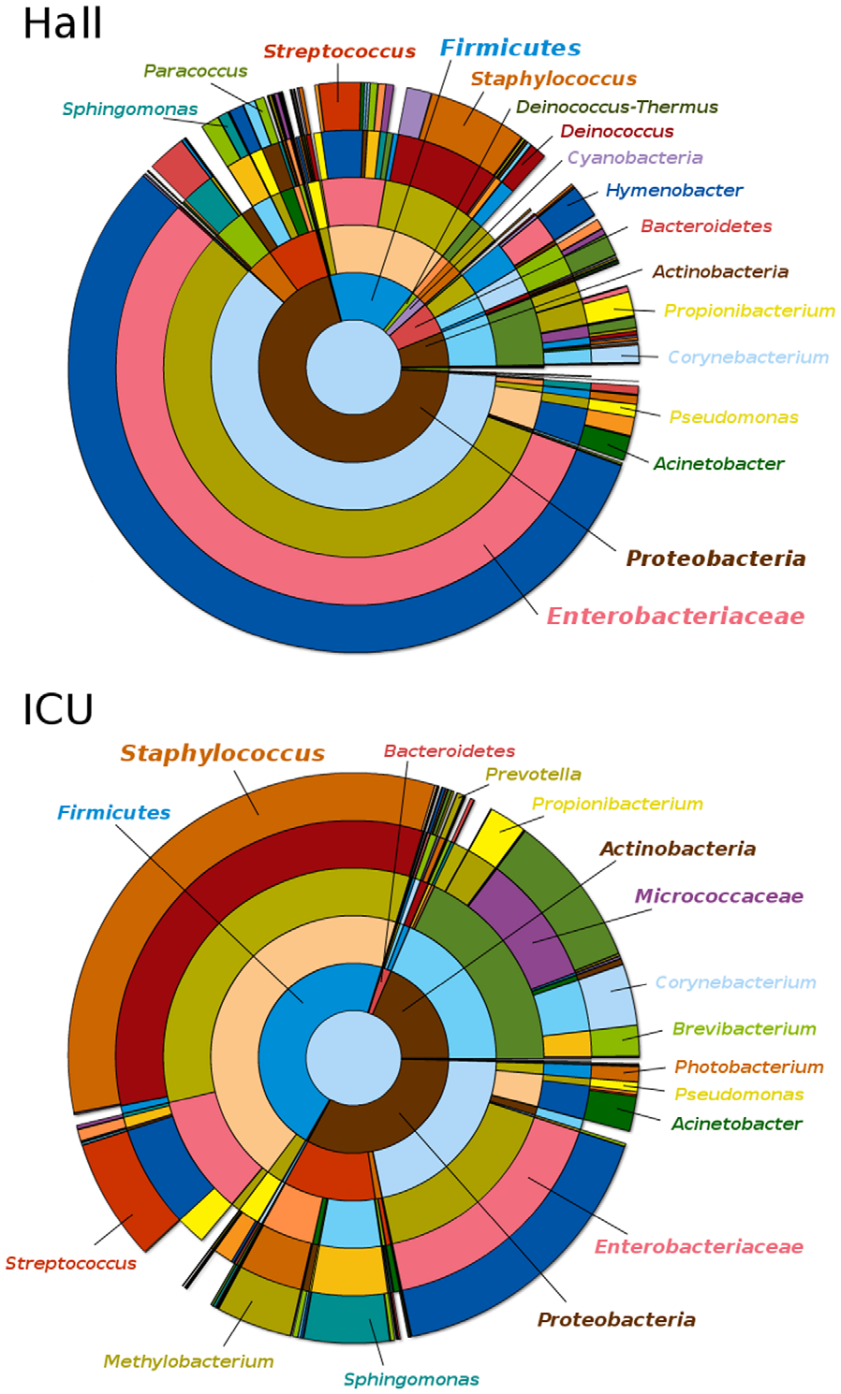
Hierarchical pie chart representing bacterial diversity assessed by pyrosequencing. Bacteria recovered from (a) Hall and (b) ICU samples during the experimental period.

Representative sequences from the 3000 OTUs detected at a distance of 0.03, corresponding to a total of 190174 sequence reads from the two samples, were classified up to genus level, as described in Materials and Methods (see Dataset A in http://www.cab.inta-csic.es/usb/HMG/. Supporting Information are interactive files therefore they have been included in an author’s website). Only 11 OTU representative sequences, corresponding to 34 individual reads, could not be classified (Table 1). Figure 3 presents a comparison of OTU relative abundances at the order level in both samples and Figure 4 shows the relative abundance of families detected in ICU and Hall samples. A hierarchical pie chart, representing the bacterial diversity recovered from ICU and Hall samples is presented in Figure 5, where the most representative genera are explicitly indicated. For more details, interactive representations can be found in Figure A, Figure B and Figure C (see http://www.cab.inta-csic.es/usb/HMG/).

To identify taxonomic nodes with differential presence in any of the two environments, observed frequencies were further normalized and relative differences (*rd*) were calculated for each taxonomic node. The statistical significance of the differences was independently assessed by computing a Chi-Square test. A total of 16 canonical phyla were detected in the present study, including both locations, and, as illustrated in Figure 5, two of them were over-represented in ICU with respect to the Hall: Firmicutes and Actinobacteria. The over representation of the phylum Firmicutes was due to the abundance of members of three orders: (i) Bacillales (*rd* = 65; *p-value* = 0), which was mainly consequence of the high abundance of members of the *Staphylococcus* genus (*rd* = 70; *p-value* = 0); (ii) Lactobacillales, mainly represented by *Streptococcus* (*rd* = 48; *p-value* = 0); and (iii) Clostridiales, mainly represented by the family Veillonellaceae (*rd* = 80; *p-value* = 3.2E-202). The over representation of the phylum Actinobacteria was consequence of the high number of members of the order Actinomycetales (*rd* = 51; *p-value* = 0) which was due to the abundant presence of members of the family Micrococcaceae (*rd* = 83; *p-value* = 0), as well as the family Corynebacteriaceae (*rd* = 55; *p-value* = 4.6E-289) and Brevibacteriaceae (*rd* = 96; *p-value* = 0). Other abundant group in ICU, although not over represented with respect to Hall, was phyllum Proteobacteria. Proteobacteria were mainly represented in ICU by the family Enterobacteriaceae and by four orders that were indeed over represented in ICU with respect to Hall; (i) Rhizobiales (*rd* = 79; *p-value* = 0), specially members of the *Methylobacterium* genus (*rd* = 93; *p-value* = 0); (ii) Sphingomonadales, mainly from the *Sphingomonas* genus (*rd* = 78; *p-value* = 0); (iii) Pasteurellales (*rd* = 63; *p-value* = 1.33E-74); and (iiii) Vibrionales (*rd* = 27; *p-value* = 3.1E-25). The phyla Proteobacteria, Bacteroidetes, Deinococcus-Thermus and Cyanobacteria were over represented in the Hall relative to ICU. The phylum Proteobacteria (*rd* = −36; *p-value* = 0) was highly represented in the Hall as consequence of the abundance of Enterobacteriales (*rd* = −54; *p-value* = 0), mainly as result of the predominant presence of unclassified members of the Enterobacteriaceae family (*rd* = −54; *p-value* = 0). Other orders of Proteobacteria over represented in the Hall were: (i) Burkholderiales (*rd* = −81; *p-value* = 0), specially the families Oxalobacteraceae (*rd* = −90; *p-value* = 0) and Comamonadaceae (*rd* = −42.73E-15; *p-value* = 0); (ii) Rhodobacteriales (*rd* = −45; *p-value* = 2.79E-72), mainly represented by the *Paracoccus* genus and (iii) Rhodospirillales (*rd* = −72; *p-value* = 2.63E-113). The phylum Bacteroidetes was over represented in the Hall mainly because of the abundance of Sphingobacteriales (*rd* = −83; *p-value* = 0), especially members of the *Hymenobacter* genus (*rd* = −83; *p-value* = 1.55E-299) and, in a lesser degree, to the order Flavobacteriales (*rd* = −64; *p-value* = 5.09E-238). The phylum Deinococcus-Thermus was basically represented by the order Deinococcales, mainly from the *Deinococcus* genus (*rd* = −88; *p-value* = 9.68E-167). Finally, the over representation of the phylum Cyanobacteria (*rd* = −87; *p-value* = 1.81E-297) was mainly due to unclassified bacteria. For more details see Table A (http://www.cab.inta-csic.es/usb/HMG/). Although not over represented with respect to ICU, Firmicutes and Actinobacteria were also significantly present in the Hall. Among Firmicutes, the most abundant orders were Bacillales, mainly represented by the *Staphylococcus* genus, and Lactobacillales, mainly *Streptococcus*. Among Actinobacteria, the most abundant order was Actinomicetales, mainly represented in the Hall by the genera *Corynebacterium* and *Propionibacterium* (for more information see Dataset A in http://www.cab.inta-csic.es/usb/HMG/). In general, the Hall sample was associated to a higher diversity than that from ICU, in agreement with richness estimations (Ace and Chao1 indices), which were approximately two times higher for the first location (Table 2). However, while the Hall was dominated by Enterobacteriaceae, plus a high number of taxa represented in moderate percentages, the ICU sample included less taxa that were more abundantly represented (i. e. Corynebacteriaceae, Micrococcaceae, Staphylococcaceae, Streptococcaceae, Sphingomodacaeae, Neisseriaceae or Pasteurellaceae), as shown in Dataset A (http://www.cab.inta-csic.es/usb/HMG/).

### Clinical Data from Patients in ICU Wards

**Figure 6:**
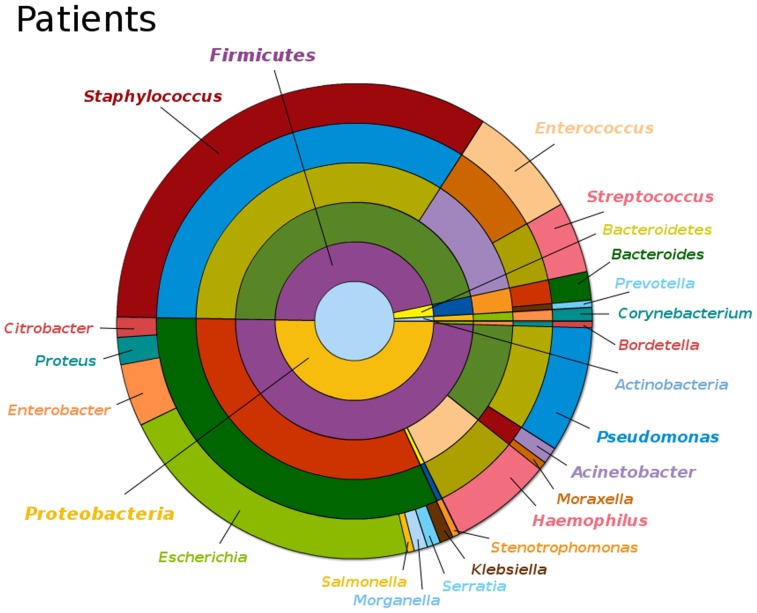
Hierarchical pie chart, representing bacterial diversity in clinical samples from ICU-hospitalized patients during the experimental period.

To recover information about infections caused in the ICU wards during the experimental period, data obtained from clinical isolates collected from ICU-hospitalized patients was analyzed. Most of the microorganisms isolated from clinical samples belonged to the phylum Proteobacteria, mainly from the classes Gammaproteobacteria and Betaproteobacteria (Figure 6 and see also Figure D in http://www.cab.inta-csic.es/usb/HMG/). Among Gammaproteobacteria, the predominant groups were the family Enterobacteriaceae, mainly members of the genera *Escherichia*, *Enterobacter*, *Proteus*, *Citrobacter*, *Klebsiella*, *Serratia*, *Morganella* and *Salmonella*, and the orders Pseudomonadales, mainly *Pseudomonas*, *Acinetobacter* and *Moraxella*, Pasteurellales, mainly *Haemophilus*, and Xanthomonadales, mainly *Stenotrophomonas*. The class Betaproteobacteria was mainly represented by the order Burkholderiales and specifically by members of the genus *Bordetella*. Other phylum detected in patient’s samples was Firmicutes, mainly represented by the orders Bacillales and Lactobacillales, with a predominance of the genera *Staphylococcus, Enterococcus* and *Streptococcus*. The phylum Bacteroidetes was also detected and represented by the order Bacteroidales, mainly *Bacteroides*, and the family Prevotellaceae, mainly *Prevotella*. Finally, the phylum Actinobacteria was represented in ICU patient samples by *Corynebacterium* members. All the microorganisms isolated from clinical samples are listed in Table B (http://www.cab.inta-csic.es/usb/HMG/).

## Discussion

Microbiology has experienced a transformation during the last 30 years that has altered the microbiologistś point of view about microbial populations. Metagenomics opened new fields of research by providing access to far more microbial diversity than has been ever viewed in the Petri dish [23]. Recently, advances in sequencing technologies have provided researchers the ability to sequence entire microbial communities in a high-throughput manner [24], [25]. The relevance of the present study lies in the characteristics of the environments analyzed: inanimate surfaces at the ICU ward and the entrance hall of the University Hospital A Coruña. ICU wards are special and confined areas where selective pressure is extreme and cleaning measurements are strict, promoting the selection of microorganisms that can develop or acquire resistance mechanisms that allow them to survive in the presence of a broad spectrum of antimicrobial agents. Patients in ICU wards are critic, weak and immunosupressed, in most cases, and therefore, even non virulent microorganisms may cause important infections leading to death. In contrast, the entrance hall of the hospital is an outward-facing and crowded place where a high diversity of people passes through and, therefore, where microbial diversity should be more related to that associated to the local human community. We consider that deep studies are needed to describe the microbial diversity associated to hospital environments, especially in ICU wards, to allow the identification of potential microorganisms causing nosocomial infections. A metagenomic study of the microbiota at hospital inanimate surfaces is difficult due to the small amount of DNA that can be recovered from the samples. This means that the genome sequences of abundant species will be well represented in a set of random shotgun reads, whereas lower abundance species may be represented by a small number of sequences. If sample size is reduced, less represented species could be missed. Pyrosequencing of 16S rRNA genes allows the deep characterization of complex microbial communities. Bacterial 16S rRNA gene contains nine hypervariable regions (V1–V9) that present considerable sequence diversity among different bacteria [26]. Combinations of conserved primer-binding sites and intervening variable sequences may facilitate genus and even species identification [27], [28]. 454-Pyrosequencing techniques by GS-FLX Titanium chemistry protocols using primers amplifying the hypervariable domains V7–V9 were successfully used for the samples used in the present study. OTU detection showed that microbiota detected in ICU was different from that in the Hall; both samples shared 644 OTUs of a total of 3000. We may extract from these data that microbial diversity differs in a confined ward subjected to selective pressure (ICU) with respect to an outward-facing and crowded hospital entrance hall, even taking into account that the lower diversity in ICU is due, in part, to the lower transit of people compared with the entrance hall, where thousands of individuals pass through a day. Besides, it is also important to remark that the area surface samples (around 200 m^2^ ICU and 50 m^2^ Hall) was not positively correlated with the diversity found. Although ICU ward is routinely strictly cleaned and supervised, a total of 15 canonical bacterial phyla were detected in this confined area in the present work. It was found that most of the microorganisms found in ICU belonged to the phyla Actinobacteria, Firmicutes, Proteobacteria and Bacteroidetes, that constituted 18.5%, 46.4%, 32.7%, and 1.4% of the bacterial community, respectively. These data agree with previous results obtained for microbial communities found in the human body [18], [29], [30], what could be explained in terms of interactions between the normal microflora living on humans and the ICU ward environment. Important differences were addressed at the order, family and genera level between ICU and Hall samples, both in terms of abundance and diversity. Data showed that although many OTUs described were detected in both hospital samples, many others could only be detected in one of the environments, this justifying the view that the two microbiotas are different. Data from clinical samples collected from in-patients correlated well with pyrosequencing data since many genera over-represented in ICU were also represented in the clinical samples (i.e. *Staphylococcus* or *Streptococcus*, which are pathogens located in any area of the skin, hands or oral flora) except for the cases of *Pseudomonas*, *Klebsiella*, *Haemophilus, Enterobacter, Citrobacter* or *Proteus*, which are common pathogens well represented in the data obtained from the clinical samples and not very well represented in ICU environment as assessed by pyrosequencing data. This could be due to the type of samples collected for pyrosequencing in this study: samples were not collected from the mechanically controlled ventilation systems, catheters or other areas of highest colonization of the ICU patients including orofaringea, perineal-inguinal, umbilical cord, axillary, cervical or lower limbs, where most of these pathogens are presumably living. Moreover, genera as *Haemophilus* or *Proteus* persist only for days in dry surfaces [22]. Families such as Micrococcaceae, Corynebacteriaceae or Sphigomonadaceae do not usually cause infectious diseases and therefore they do not appear in the clinical samples. They are, however, broadly over-represented in ICU, as assessed by pyrosequencing data, as a collection of non-pathogenic bacteria that could cause exceptional cases of opportunistic infections. Data showed that some genera over-represented in ICU were *Staphylococcus, Methylobacterium, Sphingomonas, Streptococcus or Corynebacterium. Staphylococcus*, normal inhabitant of skin and mucosae, usually infects immunocompromised humans. As an extremely versatile pathogen, this genus has developed a broad spectrum of mechanisms leading to resistance to our most powerful antimicrobial agents which could explain its presence in ICU wards. *S. aureus* is, in fact, one of the major causes of hospital acquired infection, being normal flora of ICUs [31]. The *Methylobacterium* members are methylotrophic bacteria that constitute part of the natural human foot and mouth flora. Certain species of *Methylobacterium* have been described in ICU wards causing bacteraemia in inmunosupressed patients [32]. This genus showed much more abundance in ICU with respect to the Hall and its increased presence in ICU could be explained in terms of the methanol enriched agents routinely used for cleaning. *Sphingomonas* members are metabolically versatile and can use a wide range of naturally occurring compounds as carbon source, as well as some types of environmental contaminants. Its abundant presence in ICU could be explained because this genus has a good ability to utilize a wide range of organic compounds and to grow and survive under low-nutrient conditions, including toxic compounds [33]. As an example, *S. paucimobilis* has been previously detected in hospital equipment [34] and in respiratory therapy items, bedside water bottles, sinks…etc and has been described as a cause of minor infections in humans although it is also capable of causing serious and active infections [35]. *Streptococcus,* a diverse and versatile genus that causes a broad range of diseases, is highly over-represented in ICU with respect to Hall sample. In the last years, antibiotic-resistant *Streptococcus* strains have started appearing and causing epidemics [36], [37]. *Corynebacterium* genus, normal flora of human skin, is found in a broad variety of habitats such as soil, plant material, food products and marine and animal sources. During the last years, a very considerable number of new corynebacterial species have been described as causing diseases to both healthy and inmunocomprised hosts [38]. These data show that bacteria surviving in ICU must somehow be versatile microorganisms able to adapt in low nutritional conditions, to develop resistance mechanisms, to survive in the presence of toxic compounds or to persist in dry surfaces for a long time.

Strikingly, typical microorganisms naturally living in humid environments (i.e. *Cyanobacteria, Spirochaeta* or *Thiobacillus*) have been detected in the Hall sample which could be due to the hospital location near the sea accompanied by the mild and damp climate of A Coruña.

All over the data showed that in the Hall sample there is much more biodiversity than in the ICU sample and that the most represented groups in ICU are more abundant in this ward than in the entrance Hall in many cases (i.e. *Streptococcus* or *Staphylococcus*). However, one of the most serious limitations of the present study lies on the sample size. It has to be taken into account that a bigger sample size including different hospital locations would improve or even modify the results. Even so, we could infer from the present study that the environment at ICU could be selecting for the presence of some species able to live in unfavourable conditions. We may conclude that the microbial biodiversity differs and it is reduced in a confined ward subjected to selective pressure (ICU) with respect to an outward-facing and crowded hospital entrance hall. Finally, in this study performed using new pyrosequencing technologies, we have found much more microbial diversity in hospital environments than ever seen before by classical methods. This study showed for the first time that hospital environments may act as filters selecting specific bacterial populations and creating special and confined areas where microbiota clearly differs from that present in nature.

## Materials and Methods

### Sample Collection

Samples were collected in three consecutive days of June 2009 at 8:00 a.m., before the routine cleaning, in the ICU ward (around 200 m^2^) and at the entrance hall (around 50 m^2^) of the University Hospital A Coruña, Spain. No specific permissions or ethics approval were required for these activities made at inanimate surfaces. No human samples were used. Sterile lints and gloves were used for swabbing all ICU surfaces. In the Boxes where patients are in bed, computer touch screens, monitors, drawers, infusion pumps, button panels of beds and respirator surfaces were swabbed. In the Pixies, computer touch screens and keyboards, door handles, drawers and gasometer surfaces were among the variety of sources from which samples were collected. In common areas and apparatus of the ICU wards, computer screens and keyboards, doors handles, cleaning room surfaces, medicine distributor surfaces, keyboard and electrodes of electrocardiographs, wreckers surfaces, knobs of refrigerators and drawers, microwaves and other surfaces were also cleaned with sterile lints. In the case of the entrance hall, all the accessible surfaces were combed and cleaned using sterile lints.

Lints were introduced in sterile glass beakers (pre-treated with DEPC and sterilized 30 min, 121°C, 1.1 atm) containing sterile isotonic saline solution and incubated for 2–3 h at room temperature with gentle shaking. Then, lints were wringed out and the saline solution was filtered through 0.45 µm membranes (Millipore) and centrifuged 30 min a 4000 rpm in sterile tubes to recover the particulate fraction. At this point, all pellets from ICU and Hall samples were pooled into a single sample from each location.

### Cell Wall Digestion and DNA Extraction

Both fraction pellets were resuspended in a buffer containing 20 mM Tris, pH 8, 2 mM EDTA and 1,2% Triton X-100. To digest the cell wall of the microorganisms supposedly present in the samples, the suspension was subjected to digestion using 10 mg/mL of lysozyme, 10 mg/mL of lysostaphin and 20 mg/mL of lyticase (all from Sigma-Aldrich). Digestions were performed for 1 h, at 37°C, in all cases. The Genomic Purification Kit (Promega) was then employed to recover total DNA from both samples, following the manufacturer’s instructions for each microbial type.

### 16S rRNA Amplification and Purification

In order to check the integrity of the 16S rRNA gene in the samples, oligonucleotides *16Sforw*; 5′-AGA GTT TGA TCC TGG CTC AG-3′ and *16Srev;* 5′- GAC GGG CGG TGT GTR CA-3′, commonly used to amplify the complete 16S rRNA gene (*ca*. 1300 bp) as described by Grice et al., [4] were used for PCR. The reaction mixture contained 10 µL of template (DNA extracted as described above), 5 µL of a 20 mM solution of each oligonucleotide, 3 µL of 50 mM MgCl_2_, 1 µL of a 40 mM of a mix of dNTPs, 5 µL of polymerase buffer, 0.5 µL of BioTaq polymerase (Bioline) and sterile water until 50 µL. The PCR program was as follows; one cycle of 95°C, 5 min followed by 20 cycles of 94°C, 30 s, 52°C, 30 s and 72°C, 1 min, and a final extension of 72°C, 2 min.

A segment of around 450–480 bp, containing the V7–V9 hypervariable regions of bacterial 16s rRNA gene [26] was amplified by PCR using oligonucleotides *mp16S2f* 5′-GCA TGG ITG TCG TCA GCT CGT G and *mp16S2r* 5′-ACG GIT ACC TTG TTA CGA CTT, using the primary DNA samples as template. The reaction mixture was as described above but using 1 µL of Expand High Fidelity polymerase (Roche). The PCR program was as follows; one cycle of 94°C, 2 min followed by 10 cycles of 94°C, 15 s, 52°C, 30 s and 72°C, 45 s and 20 cycles of 94°C, 15 s, 52°C, 30 s and 72°C, 2 min 25 s, with a final extension of 72°C during 7 min. Controls were performed without template.

PCR products were purified using the MinElute Purification kit (Qiagen) being the DNA finally resuspended in ultrapure nuclease-free water (Sigma-Aldrich).

The concentration and the purity grade of the samples were finally evaluated using a NanoDrop ND-1000 (Thermo scientific) and a BIOANALYZER 2100 employing the Agilent High Sensitivity DNA kit (both from Agilent Technologies, Inc. Germany).

### Pyrosequencing

Sequencing of amplicons (*ca*. 2 µg of each pure sample) was conducted in the Roche 454 Sequencing Center (Connecticut, USA) using the GS-FLX Titanium chemistry and following the manufacturer’s protocol. Both ICU and Hall samples were sequenced in the same run.

### Sequence Pre-processing and Sub-sampling

Most of the sequence processing and analyses procedures were carried out with Mothur [39], which is a package that combines a variety of tools designed to process 16S/18S rRNA gene sequence data and to characterize the diversity and structure of biological communities. Sequences were pre-processed by strictly following the Standard Operating Procedure (SOP) described by Schloss et al.[40]. In brief, pyrosequencing reads were first filtered and trimmed by imposing the following restrictions: maxambig = 0, maxhomop = 8, qwindowaverage = 35, qwindowsize = 50. This means that, those reads containing any ambiguity and those containing homopolymers longer than 8 nucleotides and, at the same time, trimmed the reads to the position in which the average quality score dropped below 35 over a 50-bp sliding window, were eliminated. Then, a non-redundant collection of sequence reads was identified, to simplify the dataset, aligned against the Core Set reference alignment of the Greengenes database [41] and screened to ensure that most of the recovered reads aligned over the same sequence space. To reduce the impact of pyrosequencing noise, the resulting reads were grouped using the pseudo-single linkage algorithm *pre.cluster*, allowing a maximal number of 1% pair wise differences, following Mothur’s implementation of the pre-clustering method [42]. Sequences were then analyzed with UCHIME, to identify and remove chimeric reads, and classified to eliminate those that could be considered contaminants (mitochondrial, plastidial and archaeal sequences). Finally, sequences were sub-sampled to normalize the number of reads from each location.

### OTU Detection

Pre-processed sequences, aligned and normalized, were used to generate an uncorrected pair wise distance matrix, which was then used to cluster the sequences using the furthest neighbor algorithm and to detect OTUs at 0.03, 0.05 and 0.1 distances. Ace and Chao1 estimates were calculated, to predict community richness, and rarefaction curves constructed, to assess sampling intensity.

### OTU Classification

OTUs detected at a genetic distance of 0.03 were classified by comparing a representative sequence from each of them against the Silva reference database [43], using BLAST to identify similar sequences [44] and the k-Nearest Neighbor algorithm to determine the consensus taxonomy from the 10 most similar sequences in the database. Sequences were classified according to the RDP6 taxonomy scheme [45]. In general, this strategy allowed the classification of sequences into taxonomic categories that were never lower than genus.

### Identification of Differentially Distributed Taxonomic Nodes

To identify taxonomic nodes for which a significant difference was observed in the number of reads associated to the ICU and Hall samples, the number of reads associated to each taxonomic node, for each of the samples, was further normalized by the total number of reads analysed classified by Mothur, after pre-processing and sub-sampling, for each sample. Then, for each taxonomic node, the relative difference *(rd)* between the numbers of reads classified in each sample was calculated and expressed as a percentage of the total number of reads classified in both samples for that taxonomic node. A Chi-Square test for homogeneity was also computed for each taxonomic node, to determine whether the normalized frequencies calculated for each sample were consistent with the null hypothesis that it is equally probable to detect a given taxon in any of the two samples.

### Clinical Data in ICU Wards

A list of microorganisms isolated from clinical samples obtained from patients staying in ICU wards during the experimental period (May, June and July of 2009) was obtained from the records of the Microbiology Department of the University Hospital A Coruña. No specific permissions or ethics approval were required for these activities.

### Availability

The pyrosequencing-derived 16S rRNA gene sequence datasets have been deposited in the GenBank short-read archive under accession number SRA046412.1.
